# To the Editor of the London Medical and Physical Journal

**Published:** 1819-07

**Authors:** R. Pew

**Affiliations:** Sherborne, Dorset


ALTHOUGH obliged to omit, in the present Number, the favours of our Correspondents, we cannot defer the insertion of the following note from Dr. Pew:

*To the Editor of the London Medical and Physical Journal.*

Sir,—The time seems at length arrived, and all the necessary facts seem now to be furnished, for the free, full, and effective, discussion of the vaccine question; and I do most sincerely hope, that this discussion may be final and incontrovertibly conclusive; and, since Dr. Forester seems kindly to have promised Dr. Bent his assistance, and it is possible that some other gentlemen may still add a few remarks on what I have said, it is my intention to put off paying my respects to Dr. Bent for some time, under the hope that I may trespass only once more on your pages; and I write this merely to assure him, that he is not forgotten, and shall not be neglected by, sir, &c. &c.

*Sherborne, Dorset; 12th June,* 1819. R. Pew.

